# Pair Nodes Clock Synchronization Algorithm Based on Kalman Filter for Underwater Wireless Sensor Networks

**DOI:** 10.3390/s21134426

**Published:** 2021-06-28

**Authors:** Xiaomeng Ni, Ting Lu, Sijia Ye, Yunsi Zheng, Pengfei Chen, Lingyu Chen

**Affiliations:** School of Informatics, Xiamen University, Xiamen 361005, China; 23320191153343@stu.xmu.edu.cn (X.N.); 15039117490@163.com (T.L.); 23320191153313@stu.xmu.edu.cn (S.Y.); 23320181154357@stu.xmu.edu.cn (Y.Z.); 23320201153981@stu.xmu.edu.cn (P.C.)

**Keywords:** synchronization, underwater wireless sensor network, K-Sync, Kalman filter

## Abstract

Time synchronization is the basis of many applications. Aiming at the limitations of the existing clock synchronization algorithms in underwater wireless sensor networks, we propose a pairwise synchronization algorithm called K-Sync, which is based on the Kalman filter. The algorithm does not need the assistance of the position sensor or the speed sensor, and the high time synchronization accuracy can be realized only by utilizing the time-stamps information in the process of message exchange. The K-Sync uses the general constraints of the motion characteristics of the sensor nodes to establish the recursive equations of the clock skew, clock offset, relative mobility velocity, and relative distance. At the same time, the time-stamps are viewed as the observation variables and the system observation equation is obtained. The K-Sync estimates the normalized clock skew and offset of the node via the Kalman filter to achieve high-precision clock synchronization between the two nodes. The simulation shows that the K-Sync has obvious advantages in the key indicators such as the estimated accuracy of clock skew and clock offset, convergence speed, etc. In addition, the K-Sync is more robust to a variety of underwater motion scenes.

## 1. Introduction

Underwater Wireless Sensor Networks (UWSNs) typically consists of power-limited terminals capable of computing and communications. UWSNs plays a key role in various applications, such as detection of seabed minerals, investigation of fishery resources, and military purposes, etc. [1,2]. Time synchronization is the basis for the collaborative execution of distributed task among a set of synchronized sensor nodes. Each sensor working in a distributed manner has its own clock. Due to the imperfect crystal oscillators [3], different clocks will drift from each other over time. Most of the applications that employ WSNs demand all of the sensor nodes to run on a common time scale, which highlights the importance of clock synchronization. In addition, data fusion, power management, node positioning, and transmission scheduling all require high-precision time synchronization [4].

The authors of [5] comprehensively study the time synchronization algorithm of terrestrial wireless sensor networks, including some widely used time synchronization protocols. However, the clock synchronization protocol for terrestrial wireless networks is not easily applicable to the underwater domain. Underwater sensor networks relying on acoustic signals to communicate have great differences compared to radio communication, due to their high speed of electromagnetic waves [6]. However, challenges brought by acoustic-based communication such as a large propagation delay, sensor node mobility, ray bending, multipath effects, limited bandwidth, and so on, also add difficulties to UWSNs [7]. Underwater communication nodes usually move with the ocean at a rate of 0.83–1.67 m/s [8], and the time-varying propagation delay caused by the slow propagation speed of the acoustic wave and nodes inherent mobility is the keys to the underwater clock synchronization algorithm. The essential work of the clock synchronization for UWSNs is to estimate the clock skew and offset [9]. Moreover, underwater nodes are resource-constrained. Therefore, it is hard for UWSNs to ensure the accuracy of clock by implementing frequent synchronization process. Existing clock synchronization methods for UWSNs mainly depend on the two-way packet exchanges between the time reference nodes and the nodes to be synchronized. Then, the clock synchronization between two nodes is realized by linear regression using the time stamps carried in sending and receiving data packets. The time synchronization of the entire network, therefore, can be realized through the clock synchronization of pair nodes.

Time synchronization for high latency (TSHL) [10], the first approach to underwater clock synchronization, proposes a two-phase synchronization algorithm. During phase I, the node clock skew is obtained by performing linear regression over multiple beacon packets; during phase II, clock offset is estimated through the two-way message exchange time-stamps of the MAC layer. However, TSHL assumes that the propagation delay is a constant, which means that nodes must be stationary, so TSHL is only suitable for static UWSNs.

MU-Sync [11], a cluster-based synchronization algorithm, uses a two-phase method to calculate the clock skew and offset employing the information obtained from message exchanges. The first phase aims to acquire the clock skew and offset by applying twice linear regression over a set of reference modem packets (beacons). The first linear regression enables the cluster head to offset the effect of long and varying propagation delay; the second linear regression is to compensate the original clock and obtain the accurate clock skew and offset. MU-Sync assumes that the propagation delay of a two-way message exchange is the same, which introduces an estimated time error. In phase 2, the synchronization phase, the so-called cluster head broadcasts the clock skew and offset to all the neighboring nodes. EMU-Sync [12] performs the second linear regression from the perspective of cluster heads and nodes to be synchronized, and takes the mean values of clock skew and offset as the estimated values, thereby outperforming the MU-Sync results.

In D-Sync, the synchronization process is started from a cluster by broadcasting a reference packet, and replied by all the neighboring nodes [13]. D-Sync employs Doppler measurements to estimate the relative speed between the cluster head and its neighboring nodes. As with MU-Sync, D-Sync is essentially a master–slave approach to handle substantial mobility. D-Sync distinguishes the propagation delays of requests and responses, which enables the synchronization accuracy to exceed the previous approaches. DA-Sync [14] applies a Kalman filter algorithm to refine the velocity estimation, which achieves marginal improvements over D-Sync.

Mobi-Sync [15], different from previous approaches, uses the temporal and spatial correlation between mobile nodes to estimate the dynamic propagation delay and the moving velocity of ordinary nodes. Mobi-Sync assumes the networks consist of three types of node: surface buoys equipped with GPS; supernodes, working as reference clocks; inexpensive ordinary nodes of low complexity. In addition, Mobi-Sync does not take into account packet loss, nor receiver node mobility while a packet travels through water. The whole process contains three phases. In phase I, the ordinary node broadcasts the synchronization request to the supernodes to acquire the time-stamps and velocity vectors. Phase II, when the ordinary nodes gather sufficient data points, linear regression is performed to calibrate the clock skew and offset. In the last phase, the calibrated results are reused for the propagation delay measurement in phase I and then reused in phase II to obtain the final accurate clock skew and offset.

Robust Opportunistic Clock Synchronization (ROCS) [16] proposes a synchronization algorithm that does not depend on the fixed synchronization process. It does not need to send specific data packets for synchronization. It only needs to insert the time-stamps in the data packets of the communication to achieve synchronization. In particular, this algorithm considers the problem of time-stamps loss caused by packet loss during underwater acoustic communication. Through matching sending and receiving time-stamp pairs correctly, the nodes can maintain synchronization even if the communication is interrupted for a long time.

Various localization algorithms have been proposed for UWSNs [17]. Based on the sensor nodes’ mobility, the authors divide localization algorithms into three categories: stationary localization algorithms, mobile localization algorithms, and hybrid algorithms. Moreover, these algorithms are all TOA-based, which usually suffer from the clock imperfections. Therefore, the time synchronization among pair nodes is required. In addition, there are some algorithms that joint clock synchronization and localization, such as STSL [18] and JSL [19]. STSL assumes that the nodes to be synchronized are within the coverage of multiple anchor nodes, equipped with a track derivation navigation system, which simultaneously achieves synchronization and positioning through periodic packet exchanges. JSL proposes a joint time synchronization and localization algorithm based on arrival time, similar to the principle of timing and positioning by GPS. Taking into account node mobility, it uses the time position information sent by four anchor nodes and the speed information provided by its own sensor to obtain the Maximum Likelihood (ML) estimate of the time and location. Modeling the stratification effect of the underwater medium with a ray-tracing approach, GN-JS [20] employs GaussNewton method to solve the original non-convex ML problem in an iterative manner, and adopts the one-way message exchange approach, which is superior to the STSL and JSL methods in both accuracy and energy efficiency.

To sum up, the majority of prior literature in the field of clock synchronization for UWSNs, except where noted, mainly depends on a series of two-way message exchanges, followed by linear regression to estimate the clock offset and drift, while ignoring the correlation between multiple message exchanges. Furthermore, most synchronization of underwater acoustic sensor networks is based on the clock synchronization between pairwise nodes. The main solutions for nodes mobility can be divided into two ways. One of the representative methods is Mobi-Sync, which utilizes the temporal and spatial correlation between mobile nodes to estimate the dynamic propagation delay and the moving velocity of ordinary nodes. However, this will introduce a large number of supernodes, so more energy will be consumed, which will also limit the scope of applications. The other way is to use Doppler information, that is, the relative speed of the ordinary nodes. Most of the existing methods improperly deal with the uncertainty of the system.

Jointly considering the regularity of sensor nodes mobility and the dynamic propagation delay, we propose a novel pairwise nodes clock synchronization approach: K-Sync. The algorithm models the system as a linear equation with the correlation of multiple bidirectional message exchanges processes, and uses Kalman filters to estimate system state variables, namely clock skew and cumulative clock offset. Moreover, the K-Sync method can achieve high precision clock synchronization merely based on the time-stamps information contained in the message exchanges, while other existing methods additionally require a sensor node’s relative speed or location information acquired by Autonomous Underwater Vehicle (AUV) [21] or some other measuring equipment.

Our main contribution is summarized as follows: the algorithm does not require additional position sensors or speed sensing device, nor a large number of super-nodes, and can achieve high-precision clock synchronization only based on the time-stamps information in a series of two-way message exchanges. According to the K-Sync algorithm, the recursive equations of the node clock skew, clock offset, relative motion velocity, and relative distance are established by using the general constraints of the motion characteristics of sensor nodes. At the same time, the system observation equation is based on viewing the time-stamps as the observation measurement. Then, the Kalman filter algorithm is used to optimize the normalized clock skew and clock offset of pairwise nodes. The simulation shows that the K-Sync algorithm has obvious advantages in some key performance indicators such as the accuracy of clock skew and offset, convergence speed and network overhead. In addition, the method is more robust to a variety of underwater motion scenes.

## 2. Theoretical Background

The main challenge of underwater sensor node clock synchronization are the dynamic propagation delay and the unsynchronized clocks between the pairwise nodes, which severely degrade the performance of global clock synchronization for the previously proposed synchronization algorithms in UWSNs. In this section, we first describe the message exchange model of the pairwise nodes. Then, the clock model and the pairwise synchronization approach are presented.

### 2.1. Overview of Clock Synchronization

**Figure 1:**
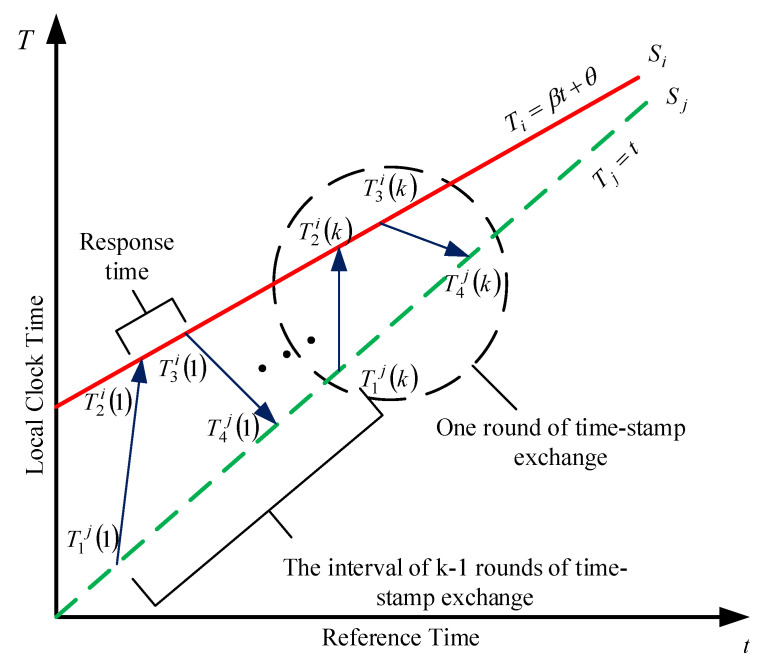
Two-way timing message exchange model.

There are typically two approaches for exchanging time synchronization messages to achieve clock synchronization in UWSNs: one-way message dissemination and two-way message exchanges. In the former, the propagation delay cannot be eliminated and the synchronization accuracy is low. Two-way message exchange, known as round-trip synchronization, is more accurate but less efficient compared to the one-way message dissemination. In order to improve the synchronization accuracy, the two-way message exchange is adopted in K-Sync, as shown in Figure 1.

In the two-way message exchange for time synchronization, assuming $S_{i}$ is the node to be synchronized and the node $S_{j}$ is the reference node, as illustrated in Figure 1, each round of time-stamp exchange includes the process of sending a synchronization message by the node to be synchronized and returning a reply message by the reference node. High precision clock synchronization is achieved through multiple rounds of time-stamp message exchanges [22]. Response time is the interval from the reference node receiving the synchronization request to returning the replied message to the node to be synchronized. The time interval between two rounds of time-stamp exchange, that is, the time interval between two synchronization requests, is initiated by the node to be synchronized. For the *k*-th round-trip message exchange process, a complete two-way message exchange is described as shown in Figure 1. $S_{j}$ initiates a round-trip synchronous message exchange with its sending time $T_{1}^{j}\left(k\right)$. After a propagation delay, $S_{i}$ receives the request message at time $T_{2}^{i}\left(k\right)$ and replies $S_{j}$ at the time $T_{3}^{i}\left(k\right)$. Then, $S_{j}$ records the reception time $T_{4}^{j}\left(k\right)$ of node $S_{i}$’s reply. According to the above steps, after the *k*-th time synchronization message exchange finishes, we can obtain *k* sets of time stamps {$T_{1}^{j}\left(1\right)$, $T_{2}^{i}\left(1\right)$, $T_{3}^{i}\left(1\right)$, $T_{4}^{j}\left(1\right)$; $T_{1}^{j}\left(2\right)$, $T_{2}^{i}\left(2\right)$, $T_{3}^{i}\left(2\right)$, $T_{4}^{j}\left(2\right)$; ……; $T_{1}^{j}\left(k\right)$, $T_{2}^{i}\left(k\right)$, $T_{3}^{i}\left(k\right)$, $T_{4}^{j}\left(k\right)$}.

### 2.2. Frequency Offset and Accumulated Clock Offset

Each individual sensor has its own clock, which is essentially a counter driven by a low-cost uncompensated crystal oscillator [23]. According to the characteristics of the crystal oscillator, the sensor node has an analog clock [24]:

(1) $$p_{i}\left(t\right)=\cos\Phi_{i}\left(t\right).$$

Here, $\Phi_{i}\left(t\right)$ is the instantaneous phase, which evolves as

(2) $$\Phi_{i}\left(t\right)=2\pi \left(f_{0}+\Delta f_{i}\right)t+\Phi_{i}\left(0\right)+\zeta_{i}\left(t\right)$$

where $\Phi_{i}\left(t\right)$ is the nominal frequency of crystal oscillator and $\Delta f_{i}$ is the frequency offset caused by hardware imperfection. $\Phi_{i}\left(0\right)$ is the initial phase, $\zeta_{i}\left(t\right)=2\pi f_{0}\sqrt{q_{i}}B\left(t\right)$ is a random process modeling phase noise. $q_{i}$ is a parameter describing degree of phase noise. B(t) represents the standard Wiener process [25]. Based on (2), the clock reading of node $S_{i}$ can be expressed as

(3) $$\begin{array}{cc} c_{i}\left(t\right) & =\frac{\Phi_{i}\left(t\right)}{2\pi f_{0}}\\ \; & =\frac{f_{0}+\Delta f}{f_{0}}t+\frac{\Phi_{i}\left(0\right)}{2\pi f_{0}}+\frac{\zeta_{i}\left(t\right)}{2\pi f_{0}}\\ \; & =f_{i}t+\theta_{i}^{0}+\sqrt{q_{i}}B\left(t\right), \end{array}$$

where $f_{i}$ is the normalized frequency, and $\theta_{i}^{0}$ represents the initial clock offset. Defining the cumulative clock offset

(4) $$\vartheta_{i}\left(t\right)=c_{i}\left(t\right)-t,$$

for any discrete observation time $t_{1},t_{2},\ldots ,t_{l},\ldots$, we can obtain

(5) $$\begin{array}{cc} \vartheta_{i}\left(t_{l}\right)-\vartheta_{i}\left(t_{l-1}\right) & =c_{i}\left(t_{l}\right)-c_{i}\left(t_{l-1}\right)-\left(t_{l}-t_{l-1}\right)\\ \; & =\left(f_{i}-1\right)\left(t_{l}-t_{l-1}\right)\\ \; & \;+\sqrt{q_{i}}\left[B_{i}\left(t_{l}\right)-B_{i}\left(t_{l-1}\right)\right]\\ \; & =(f_{i}+\sqrt{q_{i}}\frac{\left[B_{i}\left(t_{l}\right)-B_{i}\left(t_{l-1}\right)\right]}{t_{l}-t_{l-1}}\\ \; & \;-1)\left(t_{l}-t_{l-1}\right). \end{array}$$

Assuming uniform sampling($t_{l}-t_{l-1}=\tau_{0}$), the time-varing clock offset can be defined as

(6) $$\beta_{i}\left(t_{l}\right)=f_{i}+\sqrt{q_{i}}\frac{B_{i}\left(t_{l}\right)-B_{i}\left(t_{l-1}\right)}{\tau_{0}}.$$

According to the properties of standard Wiener process, we know that $\beta_{i}\left(t_{l}\right)∼N\left(f_{i},\frac{q_{i}}{\tau_{0}}\right)$. Furthermore, the time-varying clock skew of node $S_{i}$ can described as the Gauss–Markov model

(7) $$\begin{array}{cc} \beta_{i}\left(t_{l}\right) & =\beta_{i}\left(t_{l-1}\right)+\sqrt{q_{i}}[\frac{B_{i}\left(t_{l}\right)-B_{i}\left(t_{l-1}\right)}{\tau_{0}}\\ \; & \;-\frac{B_{i}\left(t_{l-1}\right)-B_{i}\left(t_{l-2}\right)}{\tau_{0}}]\\ \; & =\beta_{i}\left(t_{l-1}\right)+u_{i}\left(t_{l}\right). \end{array}$$

where $u_{i}\left(t_{l}\right)$ is the Gaussian noise with zero mean and variance $\sigma_{u}^{2}=\frac{2q_{i}}{\tau_{0}}$. Then, we can rewrite the cumulative clock offset $\vartheta_{i}\left(t_{l}\right)$ in a recursive form    

(8) $$\vartheta_{i}\left(t_{l}\right)=\vartheta_{i}\left(t_{l-1}\right)+\left(\beta_{i}\left(t_{l}\right)-1\right)\tau_{0}.$$

Substituting (7) into (8) gives

(9) $$\begin{array}{cc} \vartheta_{i}\left(t_{l}\right) & =\vartheta_{i}\left(t_{l-1}\right)+\beta_{i}\left(t_{l-1}\right)\tau_{0}\\ \; & \;+u_{i}\left(t_{l}\right)\tau_{0}-\tau_{0}. \end{array}$$

### 2.3. Sensor Node Mobility

Assuming that the reference node $S_{j}$ is completely fixed, $S_{i}$ will move within a certain range with the flow of water. In the process of two successive round-trip message exchanges, it can be assumed that the speed of the node to be synchronized relative to the standard clock reference node is constant, and the speed error is $\delta_{v}$ caused by this assumption. Then, the speed relationship of $S_{i}$ between two rounds of time-stamp exchanges can be formulated as

(10) $$v_{i}\left(k\right)=v_{i}\left(k-1\right)+\delta_{v},$$

Based on (10), assuming that $S_{i}$ moves at a constant speed, $\tau_{0}$ is the time interval between two successive message exchanges and $\delta_{d}$ is the distance error, the distance from $S_{i}$ to $S_{j}$ can be expressed as

(11) $$d_{ij}\left(k\right)=d_{ij}\left(k-1\right)+\tau_{0}v_{i}\left(k-1\right)+\delta_{d}.$$

## 3. Method

### 3.1. System State Equation and Measurement Equation Construction

Defining $x\left(k\right)={\left[\beta_{i}\left(k\right)\;\vartheta_{i}\left(k\right)\;v_{i}\left(k\right)\;d_{ij}\left(k\right)\right]}^{T}$ as the state variables of synchronizing node, combining (7), (9), (10) and (11), the state-space update equations x(k) can be described as

(12) x(k)=A(k)x(k−1)+b(k)+ω(k).

Here, the state transition matrix

$$A\left(k\right)=\left[\begin{array}{cccc} 1 & 0 & 0 & 0\\ \tau_{0} & 1 & 0 & 0\\ 0 & 0 & 1 & 0\\ 0 & 0 & \tau_{0} & 1 \end{array}\right]$$

$b\left(k\right)={\left[0\;-\tau_{0}\;0\;0\right]}^{T}$, ω(k) can be interpreted as the random disturbance in the evolution equation, that is $\omega \left(k\right)={\left[u_{i}\left(k\right)\;\tau_{0}u_{i}\left(k\right)\;\delta_{v}\;\delta_{d}\right]}^{T}$ with zero mean and the covariance matrix $E[\omega \left(k\right)\omega^{T}\left(k\right)]$.

**Figure 2:**
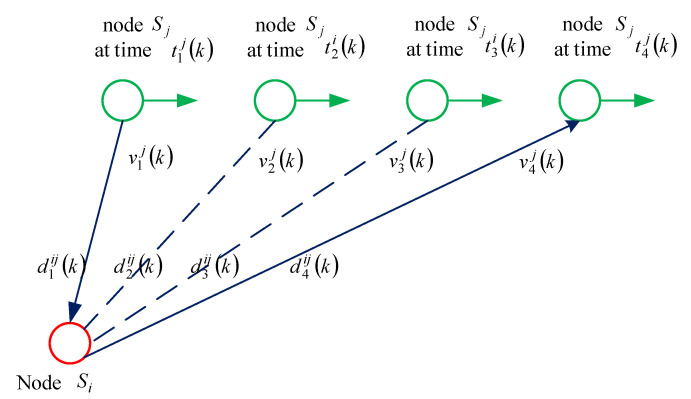
Distance representation in the *k*-th round-trip message exchanges process.

Assume that the speed of $S_{i}$ does not change over a time-stamp exchange and acoustic velocity *c* is 1500 m/s. Defining $d_{l}^{ij}\left(k\right)\left(l=1,2,3,4\right)$ corresponds to the distances between node $S_{j}$ and node $S_{i}$ in the *k*-th round of time-stamp exchanges at the reference time {$t_{1}^{j}\left(k\right)$, $t_{2}^{i}\left(k\right)$, $t_{3}^{i}\left(k\right)$, $t_{4}^{j}\left(k\right)$}, respectively, as shown in Figure 2.

Considering the *k*-th round of time-stamp exchanges process, node $S_{j}$ sends synchronization request to the reception of that message, there is

(13) $$\begin{array}{cc} t_{2}^{i}\left(k\right)-t_{1}^{j}\left(k\right) & =\frac{d_{2}^{ij}\left(k\right)}{c}\\ \; & =\frac{d_{1}^{ij}\left(k\right)+\left(t_{2}^{i}\left(k\right)-t_{1}^{j}\left(k\right)\right)v_{j}\left(k\right)}{c}. \end{array}$$

The elapsed time between node $S_{i}$ replying to the synchronization request and node $S_{j}$ receiving a reply is

(14) $$\begin{array}{cc} t_{4}^{j}\left(k\right)-t_{3}^{i}\left(k\right) & =\frac{d_{3}^{ij}\left(k\right)}{c}\\ \; & =\frac{d_{1}^{ij}\left(k\right)+\left(t_{3}^{i}\left(k\right)-t_{1}^{j}\left(k\right)\right)v_{j}\left(k\right)}{c}. \end{array}$$

Combining (13) and (14), $d_{1}^{ij}\left(k\right)$ can be eliminated

(15) $$\begin{array}{cc} \; & t_{2}^{i}\left(k\right)+t_{3}^{i}\left(k\right)-t_{1}^{j}\left(k\right)-t_{4}^{j}\left(k\right)\\ \; & =\frac{\left(t_{2}^{i}\left(k\right)-t_{3}^{i}\left(k\right)\right)v_{j}\left(k\right)}{c}. \end{array}$$

When formula (13) plus formula (14), we can obtain

(16) $$\begin{array}{cc} \; & t_{2}^{i}\left(k\right)+t_{4}^{j}\left(k\right)-t_{1}^{j}\left(k\right)-t_{3}^{i}\left(k\right)\\ \; & =\frac{\left(t_{2}^{i}\left(k\right)+t_{3}^{i}\left(k\right)-2t_{1}^{j}\left(k\right)\right)v_{j}\left(k\right)+2d_{1}^{ij}\left(k\right)}{c}. \end{array}$$

Using the reference clock $t_{l}^{i,j}\left(k\right)$ to represent the time-stamp clock $T_{l}^{i,j}\left(k\right)$. Meanwhile, considering the uncertainties brought by the demodulation of the physical layer during clock synchronization message receiving [26], a Gaussian error is superimposed on the ideal time value of sending and receiving. As a reference node, $S_{j}$ provides the time-stamps with zero accumulated clock offset. We can obtain

(17) $$\begin{array}{c} T_{1}^{j}\left(k\right)=t_{1}^{j}\left(k\right)+\delta_{1}\left(k\right),\\ T_{4}^{j}\left(k\right)=t_{4}^{j}\left(k\right)+\delta_{4}\left(k\right). \end{array}$$

As discussed above, the normalized clock skew $\beta_{i}\left(k\right)$ of node $S_{i}$ remains approximately unchanged during the *k*-th round of time-stamp exchanges procedure, so the time stamps $T_{l}^{i}\left(k\right)$ satisfy

(18) $$\begin{array}{cc} T_{2}^{i}\left(k\right) & =t_{2}^{i}\left(k\right)+\vartheta_{i}\left(k\right)\\ \; & +\left[\beta_{i}\left(k\right)-1\right]\left[d_{2}^{ij}\left(k\right)/c\right]+\delta_{2}\left(k\right),\\ T_{3}^{i}\left(k\right) & =t_{3}^{i}\left(k\right)+\vartheta_{i}\left(k\right)\\ \; & +\left[\beta_{i}\left(k\right)-1\right]\left[d_{2}^{ij}\left(k\right)/c\right]+\delta_{3}\left(k\right). \end{array}$$

The frequency offset of the node’s crystal clock can be lower than 10 ppm, that is ($\beta_{i}\left(k\right)-1)\leq 1\times 10^{-5}$), (18) can be approximated as

(19) $$\begin{array}{c} T_{2}^{i}\left(k\right)=t_{2}^{i}\left(k\right)+\vartheta_{i}\left(k\right)+\delta_{2}\left(k\right),\\ T_{3}^{i}\left(k\right)=t_{3}^{i}\left(k\right)+\vartheta_{i}\left(k\right)+\delta_{3}\left(k\right). \end{array}$$

Applying (17) and (19) to (15) and (16) gives

(20) $$\begin{array}{cc} \; & T_{2}^{i}\left(k\right)+T_{3}^{i}\left(k\right)-T_{1}^{j}\left(k\right)-T_{4}^{j}\left(k\right)\\ \; & =\left[\left(T_{2}^{i}\left(k\right)-T_{3}^{i}\left(k\right)\right)v_{i}\right]/c+2\vartheta_{i}\left(k\right)+\delta_{2}\left(k\right)+\delta_{3}\left(k\right). \end{array}$$

(21) $$\begin{array}{cc} \; & T_{2}^{i}\left(k\right)+T_{4}^{j}\left(k\right)-T_{1}^{j}\left(k\right)-T_{3}^{i}\left(k\right)\\ \; & =[\left(T_{2}^{i}\left(k\right)+T_{3}^{i}\left(k\right)-2T_{1}^{j}\left(k\right)-2\vartheta_{i}\left(k\right)\right)v_{i}\\ \; & +2d_{1}^{ij}\left(k\right)]/c+\delta_{2}\left(k\right)+\delta_{4}\left(k\right)-\delta_{1}\left(k\right)-\delta_{3}\left(k\right). \end{array}$$

Note that Equation (21) contains nonlinear terms, that is, the product term of the cumulative clock offset $\vartheta_{i}\left(k\right)$ and the relative velocity $v_{i}\left(k\right)$ of the node to be synchronized. Here, we can use the extended Kalman filter to take the partial differentiation of the system variable on the right side of Equation (21). Finally, the system observation equation can be expressed as

(22) $$\begin{array}{cc} y(k) & =H(k)x(k)+v(k)\\ \; & =[T_{2}^{i}\left(k\right)+T_{3}^{i}\left(k\right)-T_{1}^{j}\left(k\right)-T_{4}^{j}\left(k\right),\\ \; & T_{2}^{i}\left(k\right)+T_{4}^{j}\left(k\right)-T_{1}^{j}\left(k\right)-T_{3}^{i}{\left(k\right)]}^{T}. \end{array}$$

(23) $$\begin{array}{c} H\left(k\right)=\left[\begin{array}{c} \begin{array}{cccc} 0 & 2 & [T_{2}^{i}\left(k\right)-T_{3}^{i}\left(k\right)]/c & 0\\ 0 & -2v_{i}\left(k-1\right)/c & \left(T_{2}^{i}\left(k\right)+T_{3}^{i}\left(k\right)-2T_{1}^{j}\left(k\right)-2\vartheta_{i}\left(k-1\right)\right) & 2/c \end{array} \end{array}\right]. \end{array}$$

where H(k) is the observation matrix and $v\left(k\right)={\left[v_{1}\left(k\right)\;v_{2}\left(k\right)\right]}^{T}$ represents the observation noise subjecting to the Gaussian distribution with 0 mean and variance $2\sigma^{2}$, respectively.

### 3.2. Kalman Clock Synchronization Algorithm

Kalman filter is a recursive process and the estimation of system state variables can be obtained, which is an optimal minimum mean square error (MMSE) state estimator under the Gaussian assumption of both the process noise and the measurement noise. The previous state value can be used to predict the new measured value. After obtaining the latest measured value, the current system state can be estimated optimally according to the system observation equation. The Kalman clock synchronization algorithm flow is given in Algorithm 1.

**Algorithm 1:** Simulated K-Sync Algorithm1: Initialize variables
x(0) and P(0|0),(Q(0),R(0)).2: **Iteration**3: **for**
k=1,2,3……N
**do**4:  Run the time-stamps exchanges model between node $S_{j}$ and node $S_{i}$, and calculate observation matrix y(k) while obtaining {$T_{1}^{j}\left(k\right)$, $T_{2}^{i}\left(k\right)$, $T_{3}^{i}\left(k\right)$, $T_{4}^{j}\left(k\right)$}.5:  **Prediction step**6:
$$\left\{\begin{array}{c} \hat{x}\left(k|k-1\right)=A\left(k-1\right)\hat{x}\left(k-1|k-1\right)+b\left(k-1\right),\\ P\left(k|k-1\right)=A\left(k-1\right)P\left(k-1|k-1\right)A{\left(k-1\right)}^{T}+Q\left(k\right),\\ K\left(k\right)=P\left(k|k-1\right)H^{T}\left(k\right){\left(H\left(k\right)P\left(k|k-1\right)H^{T}\left(k\right)+R\left(k\right)\right)}^{-1}. \end{array}\right.$$
7:  **Update step**8:     
$$\left\{\begin{array}{c} \hat{x}\left(k|k\right)=K\left(k\right)y\left(k\right)+\left[I-K\left(k\right)\right]\hat{x}\left(k|k-1\right),\\ P\left(k|k\right)=\left[I-K\left(k\right)\right]P\left(k|k-1\right). \end{array}\right.$$
9:  **Clock synchronization**10:  Based on the calculated system variable x(k), the real clock time $\hat{t}$ can be computed
11:
$$\left\{\begin{array}{c} \hat{t}=\frac{T_{i}\left(t\right)-\theta_{i}^{0}}{\beta_{i}\left(k\right)},\\ \theta_{i}^{0}=\vartheta_{i}\left(k\right)-\left(\beta_{i}\left(k\right)-1\right)\sum_{i=1}^{N}\tau_{i}. \end{array}\right.$$
12:  Wait for the next round of two-way time-stamp exchanges, then go to step 6.13: **end for**

### 3.3. Clock Parameters Initialization

Note that before K-Sync clock parameters tracking algorithm starts, it is necessary to set the initial estimated system value ${\hat{x}}^{+}\left(0\right)$, the initial covariance matrix ${\hat{P}}^{+}\left(0\right)$ of the system variable estimation error, the covariance Q of system equation error, and the covariance R of the observation equation error.

The initial system value ${\hat{x}}^{+}\left(0\right)=E\left[x\left(0\right)\right]$, system variables $x\left(k\right)={\left[\beta_{i}\left(k\right)\;\vartheta_{i}\left(k\right)\;v_{i}\left(k\right)\;d_{ij}\left(k\right)\right]}^{T}$ contains the normalized clock skew $\beta_{i}$, accumulated clock offset $\vartheta_{i}$, relative speed $v_{i}$, and the distance $d_{ij}$ to the reference node $S_{j}$. Since the normalized clock skew estimation cannot be obtained in advance and the clock skew deviation of the two nodes is less than 10 ppm, it can be set roughly to 1. Cumulative clock offset can be calculated roughly as an initial value through a single send-to-receive two-way message exchange. $v_{i}$ can be set to 0 m/s. $d_{ij}$ can be calculated by the product of the propagation delay and the speed of sound. Although the initial value is set using the first measurement, it can be used as a rough estimate of the initial value without affecting the first Kalman filter iterative process.

The covariance of the initial estimation error of system variable is ${\hat{P}}^{+}\left(0\right)=E\left[{\hat{x}}^{+}\left(0\right)-x\left(0\right)\right]{\left[{\hat{x}}^{+}\left(0\right)-x\left(0\right)\right]}^{T}.$ Since there is no referential information for the initially normalized clock skew offset, the initial variance of the normalized clock frequency can be set to the square of the maximum normalized clock frequency of the crystal. The error of the normalized clock skew is not independent of the error of the accumulated clock offset. It can be seen from Equation (9), there is a linear relationship between them, which is related to the bidirectional interactive time interval of the node synchronization process. The correlation should be considered when setting the initial value. Similarly, there is no reliable source of information for the initial value of relative velocity. According to experience, the variance of the underwater node velocity can be set to the square of the underwater passive node maximum speed of 1.67 m/s [8]. The distance between the node to be synchronized and the reference node, as shown in (11), is not independent of each other, has a linear relationship with the relative velocity error. Considering that the initial value of the relative distance is initially estimated by the propagation delay, the initial covariance of the relative velocity can be appropriately reduced.

Q is the covariance matrix of the systematic error. It can be seen from the noise term of the system that the clock skew $\beta_{i}$ is affected by the crystal oscillator parameters; the cumulative clock offset is affected by the clock skew; the movement of the node is affected by ocean currents, and its speed change can be considered as Gaussian noise with zero mean, and the variance is affected by the actual underwater environment.

R is the covariance matrix of the observation equation. The observation value is the time-stamp generated by the synchronization message exchanges between the nodes. The domain error is the uncertainty of the underwater acoustic propagation path and the interrupt processing of the signal transmission and reception process and the delay of the bit alignment. These noises are all zero-mean Gaussian noise and are independent of each other.

### 3.4. Cramér–Rao Lower Bound Error Analysis

Cramér-Rao lower bound (CRLB) is widely used to evaluate the performance of filters and provides the best mean square error a filter can achieve. The CRLB for estimating the error at time *k* has the following form

(24) $$CRLB_{k}\overset{\Delta }{\;=}J_{k}^{-1},$$

where $J_{k}$ is Fisher information matrix

(25) $$J_{k}=E\left[-\frac{\partial^{2}\ln p\left(x_{1:k},y_{1:k}\right)}{\partial x_{k}^{2}}.\right]$$

The calculation of Fisher information matrix $J_{k}$ obeys the following recursive formula [27]

(26) $$J_{k}=D_{k}^{22}-D_{k}^{21}{\left(J_{k-1}+D_{k}^{11}\right)}^{-1}D_{k}^{12}.$$

where

(27) $$\begin{array}{c} D_{k}^{11}=E\left[-\frac{\partial^{2}\ln p\left(x_{k}|x_{k-1}\right)}{\partial x_{k-1}^{2}}\right],\\ D_{k}^{12}=E\left[-\frac{\partial^{2}\ln p\left(x_{k}|x_{k-1}\right)}{\partial x_{k-1}\partial x_{k}}\right]={\left[D_{k}^{21}\right]}^{T},\\ D_{k}^{22}=E\left[-\frac{\partial^{2}\ln p\left(x_{k}|x_{k-1}\right)}{\partial x_{k}^{2}}\right]+\\ E\left[-\frac{\partial^{2}\ln p\left(y_{k}|x_{k}\right)}{\partial x_{k}^{2}}\right]. \end{array}$$

For the following nonlinear system,

(28) $$\left\{\begin{array}{c} x(k)=A(k)x(k-1)+b(k)+\omega (k),\\ y(k)=H(k)x(k)+\nu (k), \end{array}\right.$$

For system model (28), assuming the noise is subject to a Gaussian distribution, then conditional probability density function has

(29) $$\begin{array}{c} p\left(x_{k}|x_{k-1}\right)=\frac{1}{\sqrt{2\pi \left|Q_{k-1}\right|}}\exp\left[-\frac{w_{k}^{T}Q_{k}^{-1}w_{k}}{2}\right],\\ p\left(y_{k}|x_{k}\right)=\frac{1}{\sqrt{2\pi \left|R_{k}\right|}}\exp\left[-\frac{v_{k}^{T}R_{k}^{-1}v_{k}}{2}\right]. \end{array}$$

Using (29) and the notations in (27), (27) can be written as

(30) $$\begin{array}{c} D_{k}^{11}=A_{K-1}^{T}Q_{k-1}^{-1}A_{k-1}\\ D_{k}^{12}=-A_{K-1}^{T}Q_{k-1}^{-1}={\left[D_{k}^{21}\right]}^{T}\\ D_{k}^{22}=Q_{k-1}^{-1}+H_{k}^{T}R_{k}^{-1}H_{k} \end{array}$$

Substituting (30) into (26), we can obtain

(31) $$\begin{array}{c} \begin{array}{c} J_{k}=Q_{k-1}^{-1}+H_{k}^{T}R_{k}^{-1}H_{k}-Q_{k-1}^{-1}A_{k-1}(J_{k-1}\\ \;+A_{k-1}^{T}Q_{k-1}^{-1}A_{k-1}{)}^{-1}A_{k-1}^{T}Q_{k-1}^{-1}. \end{array} \end{array}$$

The CRLB of state estimation errors is $CRLB_{k}\overset{\Delta }{\;=}J_{k}^{-1}$.

## 4. Results and Analysis

In this section, we conduct extensive MATLAB simulations to evaluate the performance of K-Sync under different underwater mobility scenarios, including a uniform motion model and a classical kinematic model (Euler Model) [28]. The simulation parameters are described later. Finally, the synchronization accuracy under different underwater scenarios is compared with that of MU-Sync [11] and D-Sync algorithms [13].

### 4.1. Simulation Setup

Unless specified otherwise, the simulation parameters are set as listed in Table 1. Transmission and reception time jitters are caused by the uncertainty of acoustic modems. Generally, the time jitter is regarded as zero-mean Gaussian noise.

### 4.2. Motion Model

Model 1: The node to be synchronized relative to the reference node can be set as 1.67 m/s [8], and then the Gaussian noise with a zero-mean variance of 0.1 m/s is superimposed on it.

**Figure 3:**
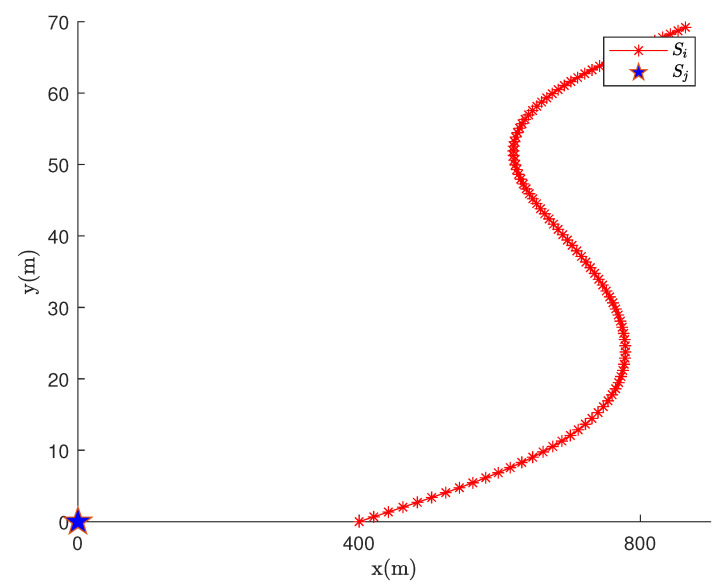
Node motion trajectory under Euler model.

Model 2: The classical Kinematic model, also known as Euler model, is used to simulate the underwater node motion. The node velocities are modeled as [28].

(32) $$\left\{\begin{array}{c} V_{x}=k_{1}\lambda v\sin\left(kk_{2}x\right)\cos\left(kk_{3}y\right)+k_{1}\lambda \cos\left(2kk_{1}t\right)+k_{4}\\ V_{y}=-\lambda v\cos\left(kk_{2}x\right)\sin\left(kk_{3}y\right)+k_{5} \end{array}\right.$$

where $V_{x}$ and $V_{y}$ denote the velocity along the *X*- and *Y*-axes, respectively. *x* and *y* correspond to the current horizontal and vertical coordinate value of the node location. $k_{1}$, $k_{2}$, $k_{3}$, λ and *v* are the random parameters changing with different environmental factors such as bathymetry and water composition. Variable $k_{4}$ and $k_{5}$ means random addictive noise. Coefficient *k* controls how fast the speed changes. According to empirical observations, we assume all random variables considered in the kinematic model are subject to normal distribution. The numerical values of these variables are set as Table 2. The movement trajectory of nodes is shown in Figure 3.

### 4.3. Number of Synchronized Rounds

**Figure 4:**
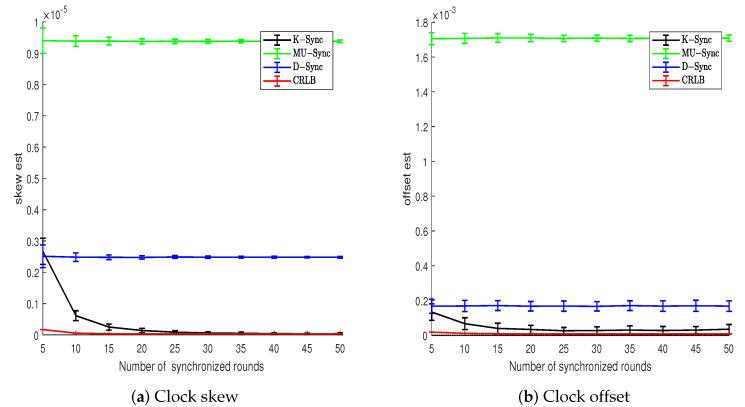
Clock parameters estimation error vs. number of synchronized rounds (Model 1).

In the scenario of uniform motion, it can be seen from Figure 4 that the clock parameters estimation error of the K-Sync clock synchronization algorithm decreases continuously with the increase in the number of two-way message exchanges, and gradually approaches its theoretical Cramér–Rao lower bound. Although the clock parameter estimation error of MU-Sync and D-Sync converges, there is always a fixed difference between them and Cramér–Rao lower bound.

**Figure 5:**
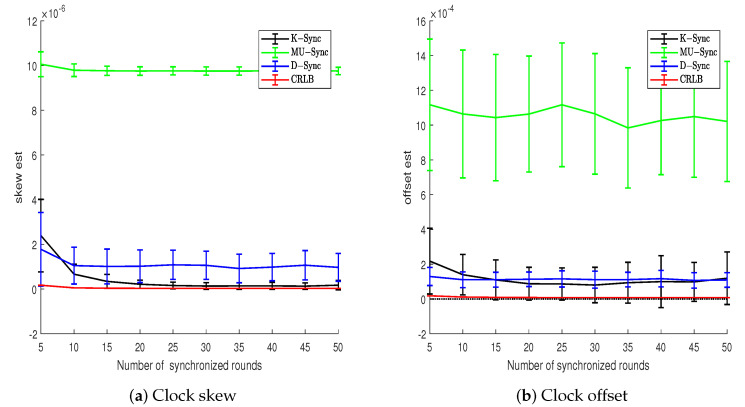
Clock parameters estimation error vs. number of synchronized rounds (Model 2).

In the scenario of Euler random motion, the motion of the node to be synchronized is relatively random. As can be seen from Figure 5, the error of clock parameters estimation of the three clock synchronization algorithms converges continuously with the increase in the number of two-way message exchange between the sender and the receiver. When the number of sending and receiving two-way messages is large, the clock parameters estimation error of K-Sync is first close to its theoretical Cramér–Rao lower, followed by that of D-Sync, and the last is MU-Sync. Compared with the scenario of uniform motion, the clock offset estimation error of the K-Sync in Euler random motion scene increases. As the velocity model of the node to be synchronized in K-Sync is not suitable for the Euler random motion, the noise covariance of the system cannot be well estimated. Moreover, K-Sync adopts fixed Q and R, which leads to its slow convergence speed, and there is a certain deviation between the convergence result and its theoretical Cramér–Rao lower bound.

### 4.4. Error after Synchronization

**Figure 6:**
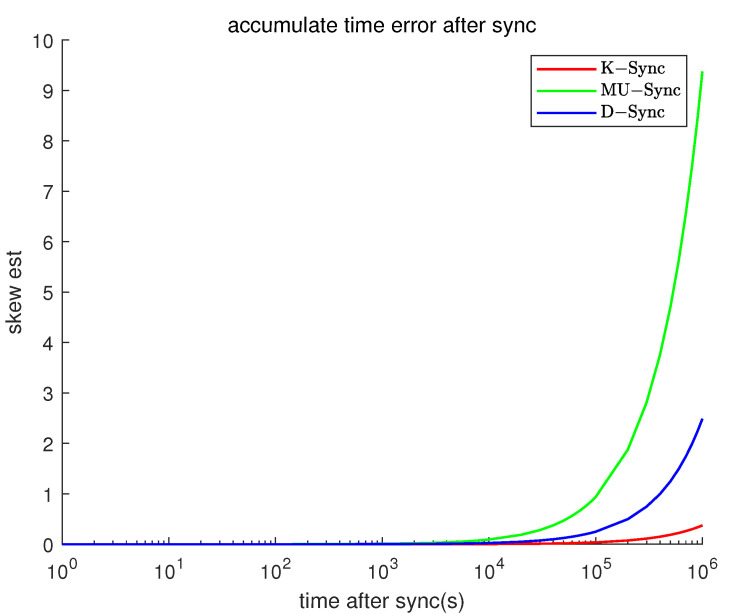
Clock error vs. time after sync (Model 1).

Figure 6 demonstrates how synchronization errors grow after time synchronization when time going by, with the methods of K-Sync, MU-Sync, and D-Sync. In this scenario, the K-Sync outperforms better than both MU-Sync and D-Sync. The performance of the MU-Sync algorithm is the worst because it is assumed that the propagation delay of each round of message exchanges is constant. Although D-Sync takes into account the relative velocities between nodes and distinguishes the different propagation delays between the request phase and the response phase, it applies the estimated speed of sound directly to the synchronization process calculation, the error is still large. The K-Sync assumes that the nodes to be synchronized are moving at a constant speed and that the speed remains unchanged during two successive message exchanges, and allows the velocity value to have some noise, which can be estimated optimally by the Kalman filter. Therefore, K-Sync can achieve high clock accuracy in uniform motion scenario.

### 4.5. Response Time and Message Exchanges Interval

**Figure 7:**
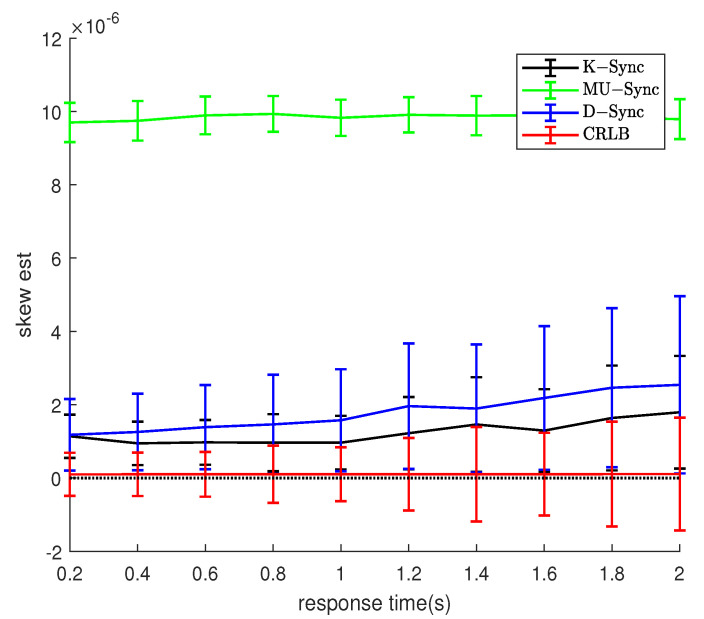
Performance with response time (Model 1).

**Figure 8:**
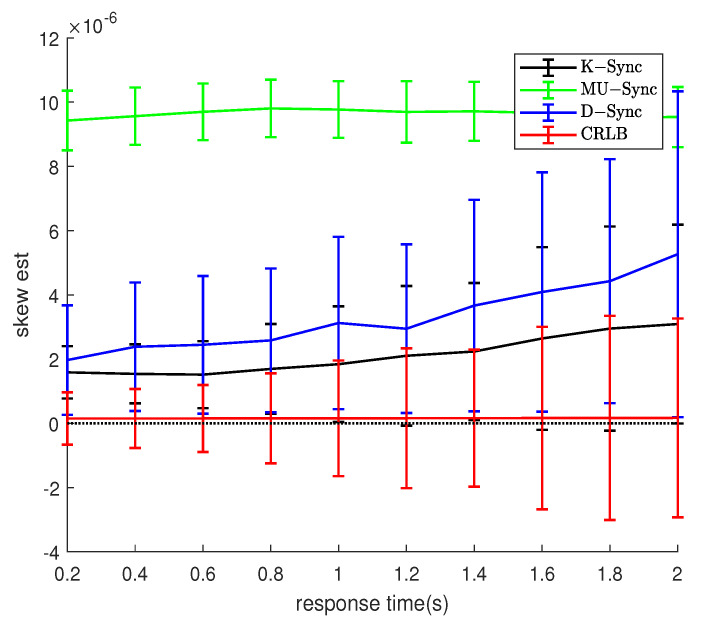
Performance with response time (Model 2).

As can be seen from Figure 7 and Figure 8, for K-Sync, MU-Sync, and D-Sync, no matter in which scenario, the estimated mean of the clock skew gradually deviates from the true value, and the variance also increases with response time increasing. The MU-Sync does not consider the mobility of the node in one round of time-stamp exchange. The longer the response time of the node is, the greater the uncertainty of the moving distance in the response time is, so the clock synchronization accuracy decreases as the response time increases. The error of the D-Sync grows the slowest because it takes into account the relative mobility of the nodes. For the K-Sync, it is clear that the error of the measurement equation of the system grows with the response time, which finally results in an increase in the error of clock synchronization.

Comparing Figure 7 and Figure 8, it can be seen that in the constant motion scenario, the synchronization error of K-Sync and D-Sync is lower than that of the random motion scenario. This is as the randomness of the motion increases, as the interaction interval increases, the estimation error of the synchronization algorithm to the speed also increases, which leads to a decrease in the synchronization accuracy. For MU-Sync, because it does not consider the relative movement between nodes, the clock synchronization errors in the two scenarios are similar.

**Figure 9:**
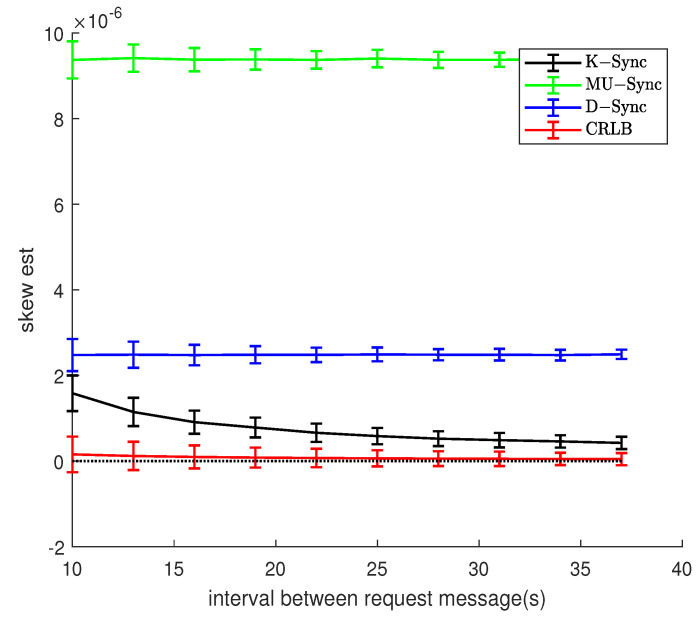
Effect of message exchanges interval (Model 1).

**Figure 10:**
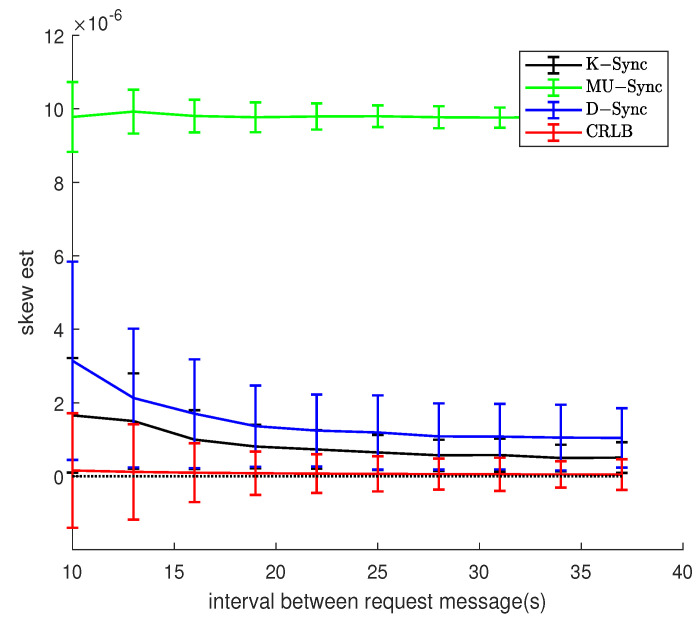
Effect of message exchanges interval (Model 2).

Figure 9 and Figure 10 show the relationship between message exchanges interval and clock skew estimation error. When the node to be synchronized is in a uniform motion scenario, the speed value does not change much during two adjacent two-way message exchanges, and the relative distance between the two nodes does not change much. The K-Sync algorithm has a better clock parameter estimation effect under the condition of a long send-interaction two-way message exchange interval.

In the random motion scenario, the greater the message exchange interval, the greater the random mobility of the node to be synchronized, the speed relevance of two adjacent sending-receiving two-way message exchanges decreases, and the system’s estimation error of speed also increases. For K-Sync, the recurrence equation of the system time state of its clock skew and accumulated clock offset takes into account the influence of the exchange interval, so as to compensate for the estimation of the clock parameters by the error in the observation to a certain extent. Therefore, in Figure 10, K-Sync is superior to the other two algorithms. However, compared with Figure 9, due to the random movement between nodes, the error variance of clock parameter estimation of K-Sync will not decrease with the increase in message exchange interval, and the stability of clock synchronization algorithm is affected by the environment.

## 5. Discussion

Efficient cooperation of sensor networks is founded on the basis of clock synchronization. The spatial correlation of nodes and Doppler information are recently employed to track the time-varying clock parameters to improve synchronization accuracy, but the correlation between the two message exchanges is ignored. This paper proposes a time synchronization algorithm called Kalman clock synchronization algorithm. The K-Sync only requires the time-stamps information in the process of message exchange without additional sensor information. By analyzing the motion properties of the sensor nodes in the synchronization process, the recursive equations of the clock skew, clock offset, relative motion velocity, and relative distance are established on the premise that the node speed between two messages bidirectional interactive is a constant. The time-stamp is viewed as observation variables, and the observation equation of the system is established. Finally, the Kalman filter method is used to obtain the optimal estimate of the clock parameters. The simulation results show that the K-Sync algorithm achieves higher accuracy when the number of message exchange is small, especially under the condition of a small two-way message exchanges interval and short response time. In addition, the K-Sync can maintain long-term accuracy of clock parameters and is robust to various sports scenes.

## Figures and Tables

**Table 1:** Simulation Parameter [10].

Parameters	Value
Initial relative distance	400
Response time	0.1 s
Message exchanges interval	10 s
Number of message exchanges	15
Time jitter variance	15 s
Acoustic velocity of sound	1500 m/s
Simulation run	2000

**Table 2:** Parameters Setting.

Setting	Value
$k_{1}$	N(π,0.1π)
$k_{2}$	N(π,0.1π)
$k_{3}$	N(0.2π,0.2π)
$k_{4}$	N(1,0.1)
$k_{5}$	N(1,0.1)
λ	N(0.3,0.03)
*v*	N(1,0.1) m/s
*k*	0.01
